# Editorial: Amino Acids of the Glutamate Family: Functions beyond Primary Metabolism

**DOI:** 10.3389/fpls.2016.00318

**Published:** 2016-03-21

**Authors:** Sakiko Okumoto, Dietmar Funck, Maurizio Trovato, Giuseppe Forlani

**Affiliations:** ^1^Department of Plant Pathology, Physiology and Weed Science, Virginia TechBlacksburg, VA, USA; ^2^Laboratory of Plant Physiology and Biochemistry, Department of Biology, University of KonstanzKonstanz, Germany; ^3^Department of Biology and Biotechnology, Sapienza University of RomeRome, Italy; ^4^Laboratory of Plant Physiology and Biochemistry, Department of Life Science and Biotechnology, University of FerraraFerrara, Italy

**Keywords:** amino acids, glutamic acid, metabolism, abiotic stresses, plant biochemistry

Amino acids in the plant body serve multiple purposes beyond being building blocks of proteins: They are major nitrogen carriers in the plants' long distance transport systems, precursors to important metabolites, nitrogen storage molecules, stress response molecules, and signaling molecules. Biochemically, the glutamine/glutamate family of amino acids (Gln, Glu, GABA, Pro, Arg) is close to the “entry point” of inorganic nitrogen (i.e., Glutamine Synthetase/Glutamate-glutamine-oxoglutarate aminotransferase [GS/GOGAT] cycle) into organic nitrogen metabolism. Furthermore, the carbon skeleton of Glu and Gln is directly connected with primary energy metabolism (TCA cycle). Biosynthesis pathways for these amino acids are regulated at multiple levels, and in turn serve to communicate developmental and environmental cues.

This Research Topic presents some new studies on physiology and biochemistry of the Gln/Glu family of amino acids, as well as summarizes our current knowledge of their roles beyond protein synthesis. Even for an “old enzyme” such as GS, new understandings emerge by transcriptome analysis and metabolite profiling; for example, García-Calderón et al. found that, under stress conditions, a defect in GS2 activity affects phenolic metabolism in *Lotus japonicus*. These data indicate that the activity of GS impacts metabolism beyond amino acids and related primary metabolites. Also, not all isoforms of GS in plants are equally regulated by the same type of post-translational modifications. Recent studies on GS complexes from *Medicago truncatula*, which involve multiple regulatory molecules and are heavily compartmentalized, are summarized by Seabra and Carvalho. The function of Glu as the direct precursor for chlorophyll and heme biosynthesis in plants has recently been reviewed by Brzezowski et al. (2015) and is not included in this Research Topic.

GABA, which is synthesized from Glu by a single decarboxylation reaction, plays a versatile role in plants and, together with proline (see below), is one of the most common osmolytes accumulated by plant cells in response to stress. Accumulation of GABA also occurs under non-stress condition in specific tissues, e.g., growing tomato fruits, and functions in defense or taste development are being suggested (Takayama and Ezura). GABA is further catabolized within the mitochondria through a two-step pathway called the GABA shunt, which bypasses a part of the TCA cycle. The pathway plays a crucial role in carbon metabolism, especially at specific growth stages or under adverse conditions such as low light and water stress. Hence, as thoroughly discussed in Michaeli and Fromm, GABA levels could function as the key signal connecting metabolism to developmental or environmental responses. Corroborating this notion, a study using a loss-of-function mutant of a GABA transporter provides evidence that GABA transport acts in regulating C/N balance signaling in Arabidopsis (Batushansky et al.). In some specific cases, the function of GABA as signal has been established, such as its role in pollen tube guidance (Biancucci et al.). In this context, a recent work showed that GABA acts as a genuine signaling molecule by directly modulating a membrane malate channel, ALMT (Ramesh et al., 2015). It remains to be seen whether some of the functions discussed in this Research Topic are mediated by the modulation of membrane transporter activity.

Another amino acid playing a pivotal role in stress responses is proline. It is well established that proline is accumulated in specific tissues at certain developmental stages such as in pollen grains (Biancucci et al.; Kavi Kishor et al.) and under stress conditions (i.e., salt, drought, pathogen attack) as a result of transcriptional and post-translational regulations. The biochemical mechanisms of the protective functions of proline in stress responses are starting to be understood. The role of P5C, a toxic intermediate in proline metabolism, in the production of reactive oxygen species (ROS) is reviewed by Qamar et al. Likewise, the role of proline-derived ROS production during senescence is also discussed in detail by Zhang and Becker.

Although the biochemical pathways of proline biosynthesis and catabolism are known, some aspects of enzymatic regulations had never been characterized in plants. In this Research Topic, Forlani et al. (number 565) report an extensive structural and biochemical characterization of δ^1^-pyrroline-5-carboxylate reductase (P5CR), and conclude that NADPH is the likely co-factor *in vivo*, and that stress-induced variations in redox and ion homeostasis could profoundly affect the enzymatic activity. A refined crystal structure and analysis of P5CR from *Medicago truncatula* indicated that these are common features of plant P5C reductases (Ruszkowski et al., 2015). Some of the properties, such as co-factor preferences, could be inferred by comparing the primary sequence between P5CRs from different organisms, suggesting evolutionarily conserved domains (Forlani et al., number 567). Post-translational mechanisms also seem to be important in regulating proline degradation, in particular δ^1^-pyrroline-5-carboxylate dehydrogenase (P5CDH) activity. The activity of rice P5CDH was strongly dependent on factors such as cation concentrations, co-factor availability, redox status, and free arginine pools, while modulation of transcript levels contributed little to the regulation of total enzyme activity, underscoring the importance of detailed biochemical analyses (Forlani et al., number 591). Another level of complexity is added by interactions between multiple steps that control proline levels; using a detailed metabolite and transcript analysis in a *p5cdh* mutant, Rizzi et al. showed that P5CDH modulates the flux through the pathways of proline biosynthesis by influencing the expression levels of δ^1^-pyrroline-5-carboxylate synthetase.

In addition to its metabolic roles, proline and its derivative, hydroxyproline, are also major constituent of cell wall proteins such as extensins. These polypeptides have cross-linking capability as cell wall components, and are regulated by developmental and environmental cues. Kavi Kishor et al. discuss the function of proline/hydroxyproline rich proteins in the cell wall in relation to proline metabolism.

Another group of amino acids within the Gln/Glu family, also linked to Pro metabolism through the common intermediate P5C, is composed of Arg and Orn. Arg biosynthesis is tightly regulated in plants by post-transcriptional modifications, feedback mechanisms, and interactions with signaling proteins to ensure appropriate Arg production for protein synthesis and nitrogen storage. As a link to signaling and stress defense, the conversion of Orn or Arg to polyamines is well documented, while the production of Nitric Oxide (NO) from amino acid precursors is mechanistically still unresolved in plants (Winter et al.). In addition, Orn is suggested to play a direct role as a signaling molecule, too. Increased consumption of Orn by overexpression of mouse Orn decarboxylase in Arabidopsis caused increased levels of polyamines while amino acid levels were not strongly altered, indicating that nitrogen assimilation and allocation to different metabolic pathways may be regulated by Orn. Interestingly, the regulatory changes seemed to happen predominantly at the post-transcriptional level (Majumdar et al.).

So where does the research on the Gln/Glu family of amino acids go from here? Although the scientific community has learned a lot in the past decade due to an increased amount of genetic resources and technological advances in mRNA profiling, some critical details are still unknown. For example, the metabolic pathways of the Gln/Glu family of amino acids involve three major cellular compartments (cytosol, mitochondria, and chloroplasts) that differ in critical parameters such as metabolite and ion concentrations and redox status. The biochemical studies presented in this issue clearly demonstrate that the conditions specific to the given cellular compartment need to be considered carefully, and that transcriptome data alone does not reflect the pathway activity correctly. Additionally, the transport mechanisms and kinetics between the compartments need to be characterized. Such efforts would eventually provide a predictive model of amino acid flux, which will inform us of critical factors behind phenotypically important traits. For example, a perspective article presented in this issue (Bhaskara et al.) proposes that the classic view of “more proline is better for stress resistance” might not be correct, and rather, it might be the flux through the pathway that matters. Such a hypothesis can be examined with a predictive flux model.

Another exciting recent development are the roles that the Gln/Glu family of amino acids play in signaling, either serving as signaling molecule themselves or as precursors. It is well established that the addition of Gln/Glu induces a massive transcriptional change in plants (Gutiérrez et al., 2008), and amino acid imbalance causes stress responses in plants (Pilot et al., 2004; Hirner et al., 2006; Liu et al., 2010; Yu et al.). Also Pro has recently been identified as a signaling molecule regulating plant growth and development (Wang et al., 2014; Biancucci et al., 2015). What exactly is being sensed to initiate such responses? Are amino acids being sensed by the mechanisms we now know, such as glutamate-receptor like proteins (Price et al., 2012; Forde and Roberts, 2014) or ALMT (Ramesh et al., 2015), or are there additional mechanisms for recognition? Genetic tools to dissect amino acid sensing mechanisms, such as the ones presented by Yu et al. will help discern the key players in amino acid sensing and signaling pathways.

## Author contributions

All authors listed have made substantial, direct, and intellectual contribution to the work, and approved it for publication.

## Funding

GF acknowledges support from AGER Foundation in the frame of the RISINNOVA project, grant # 2010-2369. SO acknowledges support from NSF, grant MCB-1052048.

### Conflict of interest statement

The authors declare that the research was conducted in the absence of any commercial or financial relationships that could be construed as a potential conflict of interest.
